# Mapping current trends and hotspots in myasthenia gravis from 2003 to 2022: a bibliometric analysis

**DOI:** 10.3389/fneur.2023.1320344

**Published:** 2023-12-28

**Authors:** Yukun Tian, Qiqi Shen, Siyang Peng, Linghao Meng, Ruiying Fang, Anni Xiong, Shaohong Li, Yajing Yang, Weiqian Chang, Jinxia Ni, Wenzeng Zhu

**Affiliations:** ^1^Department of Acupuncture, Guang’anmen Hospital, China Academy of Chinese Medical Sciences, Beijing, China; ^2^Treatment Center of Traditional Chinese Medicine Bo’ai Hospital, China Rehabilitation Research Center, Beijing, China; ^3^Department of Traditional Chinese Medicine, Yuyuantan Community Health Center, Beijing, China; ^4^Department of Acupuncture, Guang’anmen Hospital, Chinese Academy of Traditional Chinese Medicine Ji’nan Hospital (Ji’nan Hospital of Traditional Chinese Medicine), Jinan, Shandong, China; ^5^Department of Acupuncture, Dongzhimen Hospital of Beijing University of Traditional Chinese Medicine, Beijing, China

**Keywords:** myasthenia gravis, bibliometric analysis, VOSviewer, CiteSpace, citations, keywords

## Abstract

**Introduction:**

Research on myasthenia gravis (MG) has undergone rapid development in recent years. This article aimed to elucidate the characteristics of MG publications over the past 20 years and analyze emerging trends using bibliometric methods.

**Methods:**

Information on MG articles was obtained from the Web of Science Core Collection and stored in Excel for quantitative analyses. Bibliometric analyses were performed using CiteSpace and VOSviewer to visualize publications according to countries/regions, institutions, journals, and authors.

**Results:**

A total of 3,610 publications were included in the analysis. The USA had the highest number of publications (NP) and H-index. Among the institutions, the University of Oxford had the highest NP, followed by the University of Toronto and Duke University. Close cooperation was observed among countries and institutions. The most productive author was Renato Mantegazza, followed by Jan J. Verschuuren, and Amelia Evoli. *Muscle & Nerve* published the most articles on MG, followed by the *Journal of Neuroimmunology* and *Neuromuscular Disorders*. The keyword with the highest strength is “neuromuscular transmission,” followed by “safety” and “rituximab.” Co-citation analysis includes 103 publications cited at least 65 times, categorized into four clusters. Additionally, 123 keywords cited more than 40 times were analyzed and divided into five clusters.

**Conclusion:**

This bibliometric analysis shows the framework of research over the past 20 years by mapping the scholarly contributions of various countries or regions, institutions, journals, and authors in MG. The analysis also explores future trends and prospective directions, emphasizing individualized treatment based on subtypes, novel immunotherapeutic approaches, and thymectomy.

## 1 Introduction

Myasthenia gravis (MG) is an autoimmune disease characterized by skeletal muscle weakness and fatigability. It is caused by antibodies directed against proteins at the neuromuscular junction, including the acetylcholine receptor (AChR), muscle-specific kinase (MuSK), lipoprotein-related protein 4 (LRP4), and the postsynaptic membrane (1). The prevalence of MG is estimated to be 100 to 350 cases per 1 million people, with increasing trends likely due to improved diagnostics, aging populations, and the number of systematic epidemiological studies conducted over the past decade (2–4).

Bibliometric analysis has become an internationally recognized tool since 1958 that not only contributes to important decisions about government budgets (5), but more significantly, also helps understand new research areas in rapidly advancing fields. By analyzing the relationships between journals, citations, keywords, and scholars, it enables the creation of visual maps to represent publications and academic networks. MG research has expanded substantially in recent years. The aim of this study was to use bibliometric techniques to characterize patterns in MG publications over the past 20 years and elucidate trends in this growing area of inquiry.

## 2 Methods

### 2.1 Data acquisition and search strategy

**Figure 1:**
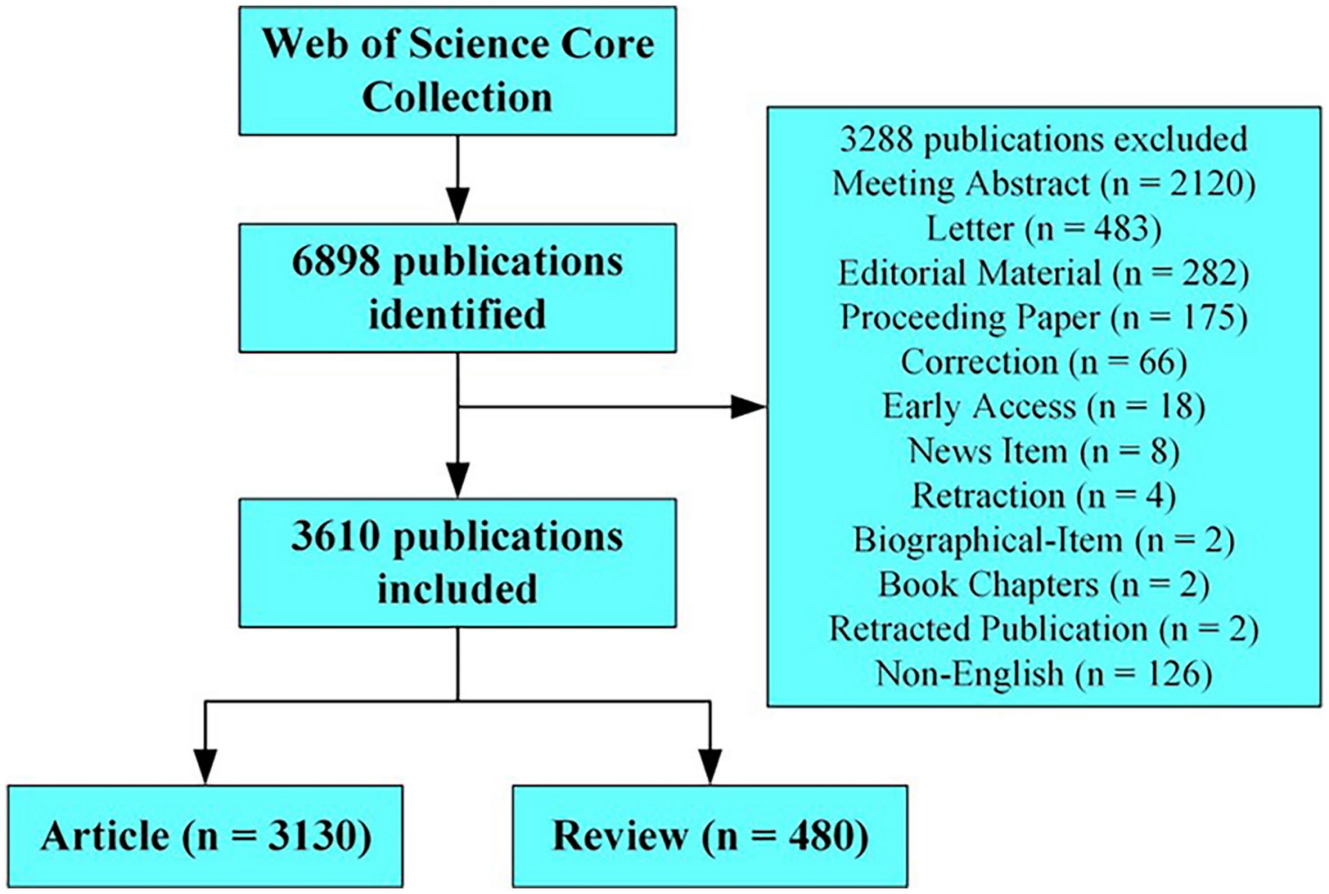
Flowchart of literature screening.

Data were collected from the Web of Science Core Collection, and the search period was set between 1 January 2003 and 31 December 2022. A literature search was conducted on 9 June 2023. The search terms were as follows: ts = “myasthenia gravis” (ts = topic), and 6,898 publications were identified. The articles and reviews in English were analyzed after removing the meeting abstracts, letters, editorial materials, proceedings, corrections, early accesses, news items, retractions, biographical items, book chapters, and retracted publications. A total of 3,130 articles and 480 reviews were included in the analysis. The collected data included the journal titles, authors, keywords, countries or regions, institutions, and cited references, and the downloaded data were screened by S.Y. Peng to identify the relationships on the topic. The strategy for accessing and searching for articles is shown in Figure 1.

### 2.2 Data analysis

A quantitative analysis of the number of publications (NP) and citations (NC) in the most productive countries, authors, journals, and affiliations was conducted using Microsoft Office Excel 2013. VOSviewer (Centre for Science and Technology Studies, Leiden University, Leiden, the Netherlands) is a computer program used to create scientific maps for co-citation analysis, co-occurrence of keywords, and co-authorship analysis of authors, affiliations, and countries (6). CiteSpace was used to visualize and analyze bursts of references and keywords (7).

## 3 Results

### 3.1 Annual trends in the number of publications

**Figure 2:**
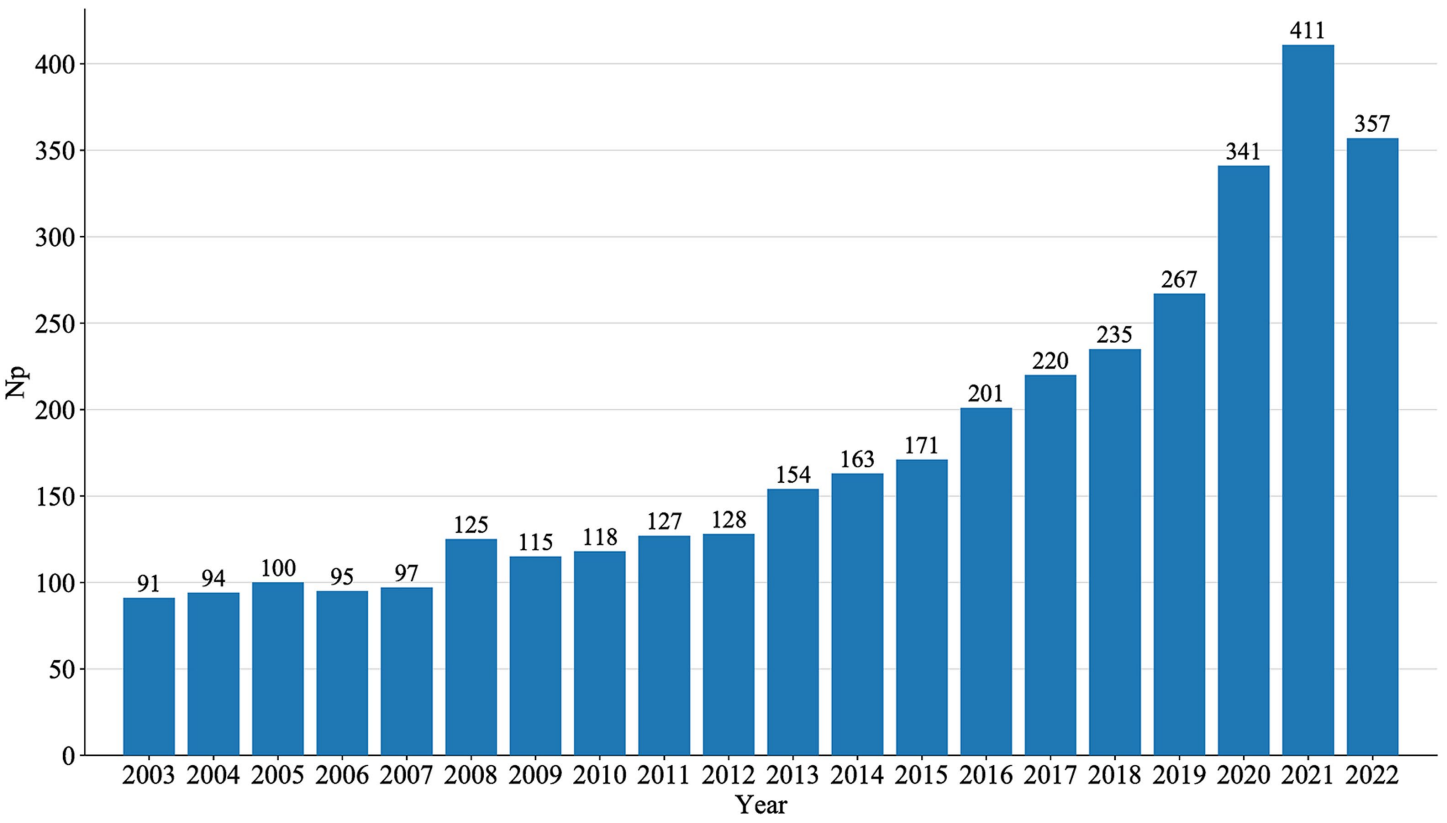
Current status of global publications.

An annual analysis of NP directly reflects trends in a specific research field over a given period. The annual publications of MG generally showed a steady growth trend, peaking in 2021, as shown in Figure 2.

### 3.2 National research status and international cooperation

**Figure 3:**
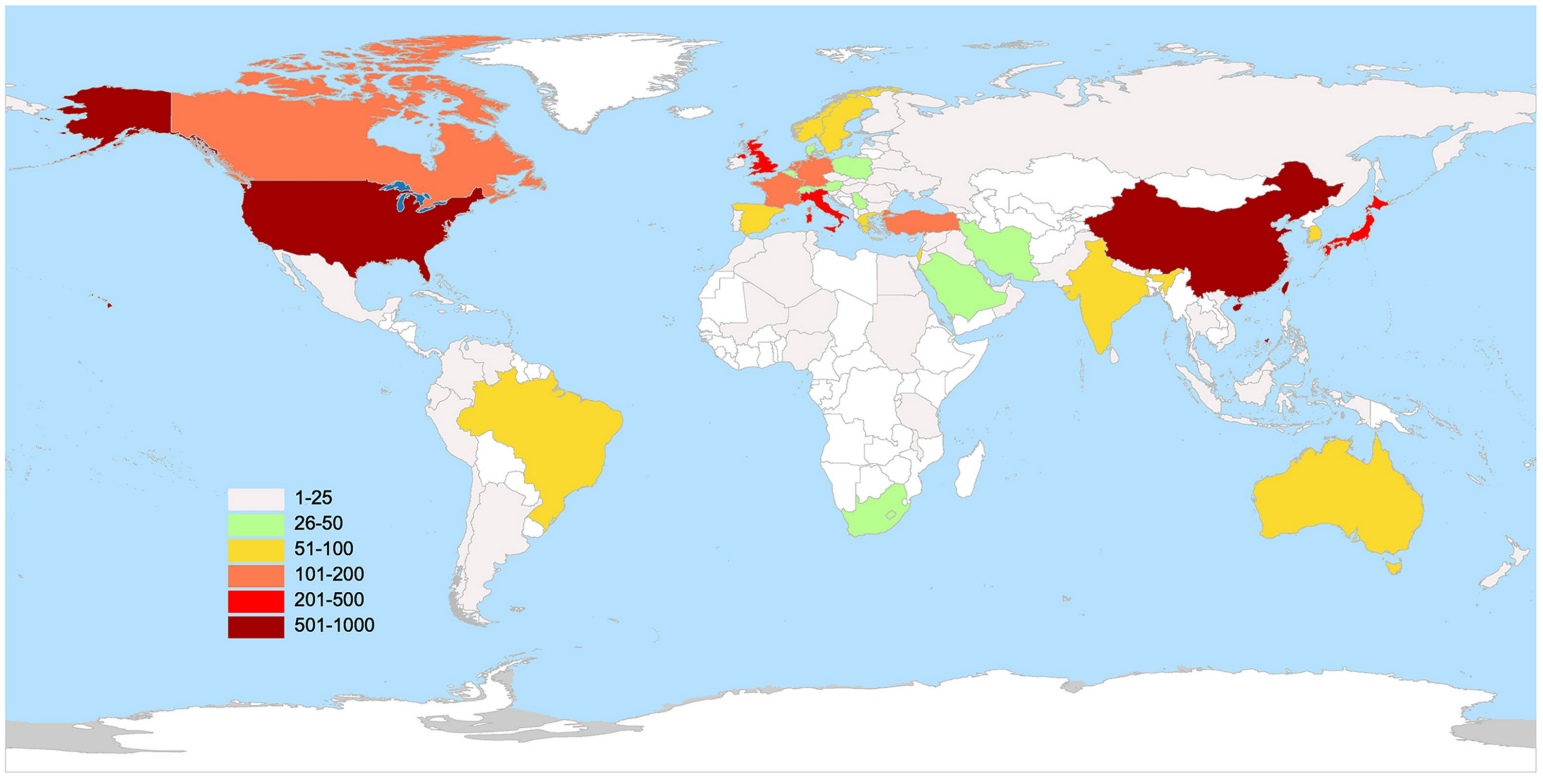
The geography distribution map manifested the number of publications in distinct countries.

**Figure 4:**
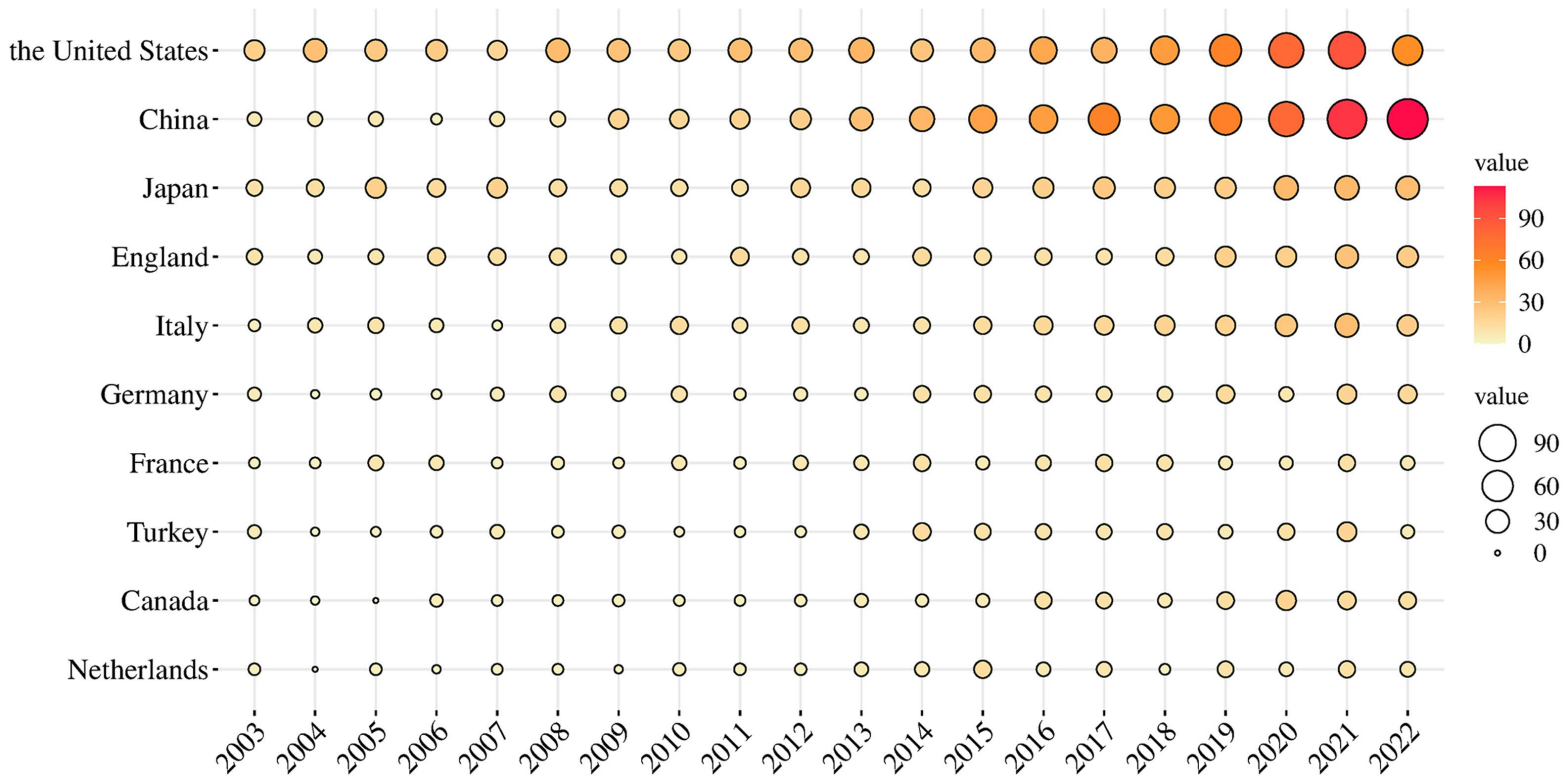
The bubble chart of yearly publications of countries.

Figure 3 displays the world distribution map based on the NP in different countries. In this figure, the depth of the color represents the magnitude of NP. The top 10 countries in NP are presented in Table 1. It is evident that the USA had the highest NP over the past two decades in MG research, followed by China, Japan, the UK, and Italy. The USA had the highest number of total citations after removing self-citations, followed by the UK, Italy, China, and Japan, with H-indices of 68, 49, 45, 37, and 38, respectively. H-indices reflect the general quality of a country’s literature. Figure 4 present a bubble chart of the yearly publications of the top 10 countries in NP is shown in Figure 4, demonstrating an annual increase from 2003 to 2022. The USA led in yearly publications until 2014, after which China took the lead and continued until 2022.

**Table 1:** The top 10 most productive countries.

Rank	Country	Np	Nc	H-index	Average citations
1	China	747	7,736	37	10.36
2	the United States	767	20,097	68	26.20
3	Japan	375	7,369	38	19.65
4	Italy	269	8,236	45	30.62
5	England	271	9,220	49	34.02
6	Germany	169	5,503	38	32.56
7	France	142	4,898	40	34.49
8	Canada	135	3,939	29	29.18
9	Turkey	142	1787	21	12.58
10	Netherlands	116	5,607	38	48.34

**Figure 5:**
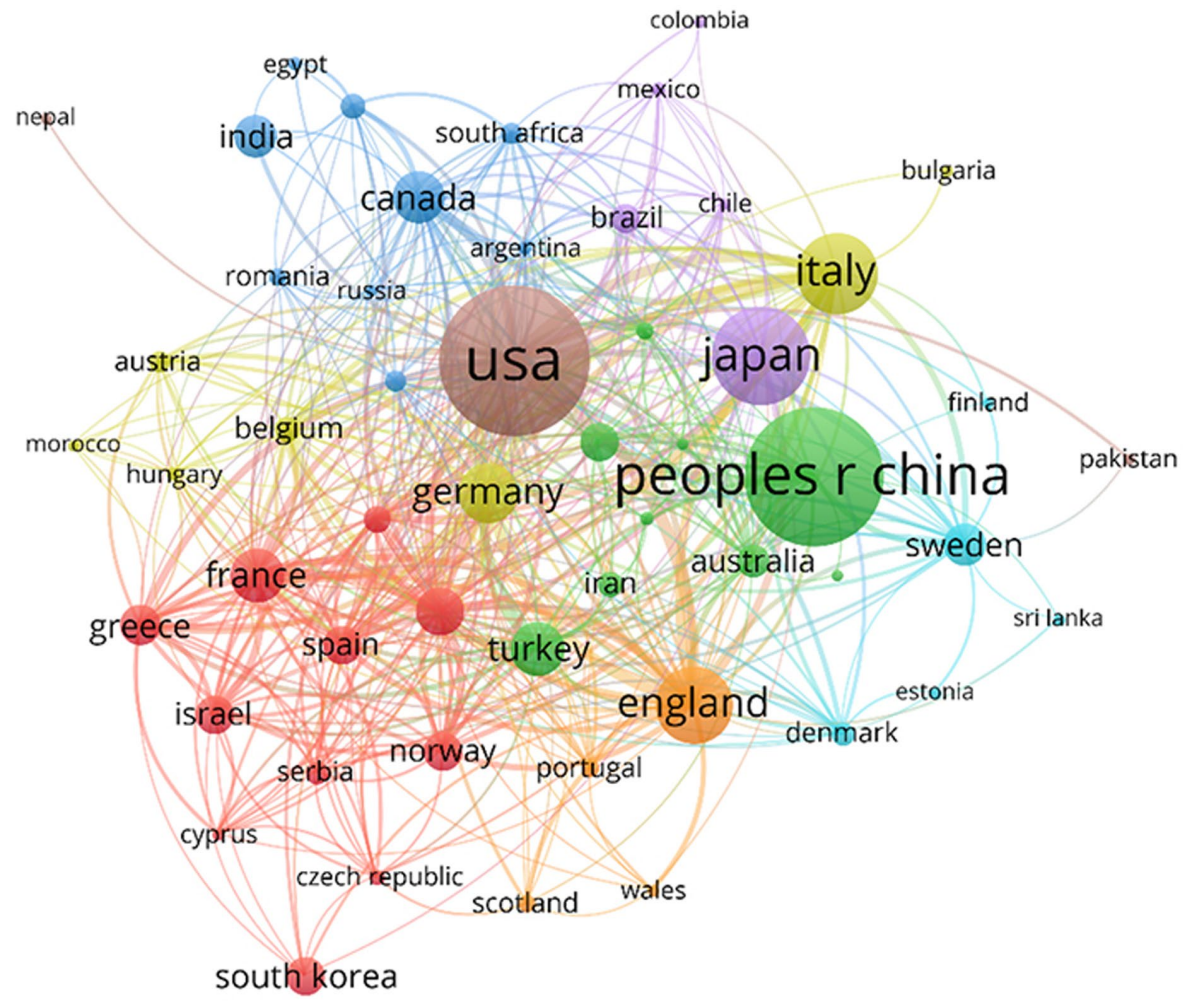
Cooperations among countries or regions.

In the co-authorship analysis (Figure 5), 55 countries with a minimum of five publications were included. Countries were divided into eight clusters of different colors. Bold lines between countries indicate closer collaborations, and the node size reflects the centrality and influence of the country on associations. Owing to the diversity of scientific research foundations and resources in other countries or regions, a specific collaborative network structure was observed at the national or regional scale. According to this map, cooperation between most countries was close. The USA, China, Japan, the UK, Italy, France, and Canada were the central countries in cooperation, with the USA being the most influential.

### 3.3 Analysis of institutions

**Figure 6:**
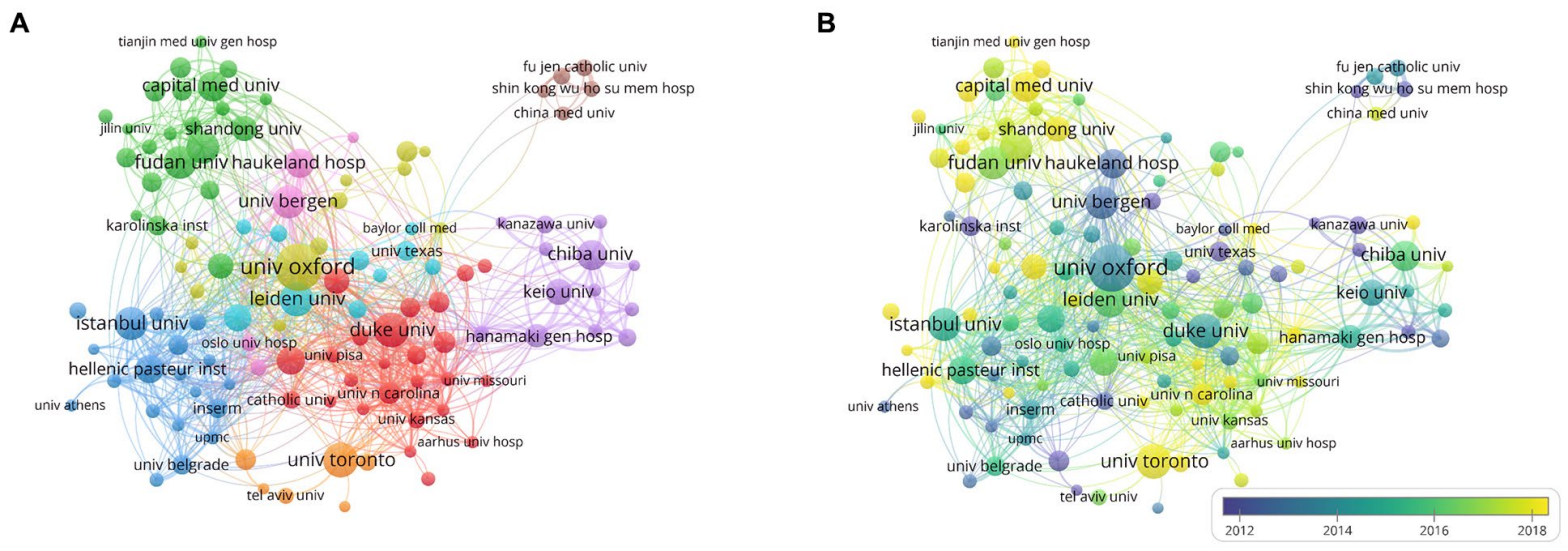
Network visualization of cooperations among institutions. **(A)** Visualization of institutions divided into nine clusters. **(B)** Visualization of the institutions network according to the average years of publication.

The top 10 institutions in the total NP in this research area are listed in Table 2. Oxford University had the highest NP, with 94 publications, followed by the University of Toronto (62) and Duke University (61). One hundred sixty institutions with 10 or more publications were included in VOSviewer for co-authorship analysis. As shown in Figure 6A, the affiliations were divided into nine clusters. Close collaborations among Oxford University, Leiden University, and Duke University were apparent. Meanwhile, Fudan University, Istanbul University, University of Toronto, University of Bergen, and Chiba University were the centers of the collaboration clusters. However, because to geographical distance, a lack of cooperation between several institutions from different countries was observed, whereas cooperation was closer within each cluster because the institutions in a cluster were from the same countries. We also visualized the time overlay for the co-authorship institutional analysis using VOSviewer. As shown in Figure 6B, the institutions were divided into different colors according to the average year of publication; the institutions in yellow were active later than those in blue. The University of Toronto, Capital Medical University, and Shandong University recently appeared more in cooperation, indicating that they were more active in MG research and full of potential in international cooperation.

**Table 2:** The top 10 most productive institutions.

Rank	Affiliations	Country	Np	Nc	H-index	Average citations
1	University of Oxford	UK	120	6,073	38	50.61
2	Udice—French Research Universities	France	109	4,123	36	37.83
3	University of Texas System	the US	84	2,250	23	26.79
4	Catholic University of the Sacred Heart	Italy	78	4,144	28	53.13
5	IRCCS Policinivo Gemelli	Italy	77	4,127	28	53.60
6	Institut National de la Sante et de la Recherche Medicale Inserm	France	72	3,018	29	41.92
7	Sorbonne University	France	70	2,559	29	36.56
8	University of Toronto	Canada	70	1,668	21	23.83
9	Leiden University	Netherlands	67	3,893	27	58.10
10	Duke University	the US	64	4,615	34	72.11
11	IRCCS Istituto Neurologico Besta	Italy	64	2,647	28	41.36
12	University of Bergen	Norway	64	3,937	32	61.52
13	University of California system	the US	64	2,872	23	44.88

### 3.4 Analysis of journals

The influence of a journal is expressed not only by the NP, but also by the NC. In Table 3, we summarized the top 10 journals with the highest NP in MG research. The journal with the highest NP was *Muscle & Nerve* (*n* = 224), followed by the *Journal of Neuroimmunology* (*n* = 117) and *Neuromuscular Disorders* (*n* = 98). Nine of the top 10 journals had an impact factor exceeding 3 and were mostly focused on neuromuscular diseases, neuroimmunology, and neurology.

**Table 3:** The top 10 most productive journals.

Rank	Journals	IF (2022)	Np	Nc	H-index	Average citations
1	Muscle & Nerve	3.852	224	5,755	39	25.69
2	Journal of Neuroimmunology	3.221	177	3,252	32	18.37
3	Neuromuscular Disorders	3.538	98	1,253	18	12.79
4	Frontiers in Neurology	4.086	90	637	13	7.08
5	Journal of the Neurological Sciences	4.553	82	1,520	22	18.54
6	European Journal of Neurology	6.288	72	1,697	24	23.57
8	Neurological Sciences	3.830	68	649	15	9.54
7	Neurology	11.800	67	4,383	35	65.42
9	Journal of Neurology	6.682	64	1746	24	27.28
10	Journal of Clinical Neuroscience	2.116	45	461	12	10.24

### 3.5 Author analysis

**Figure 7:**
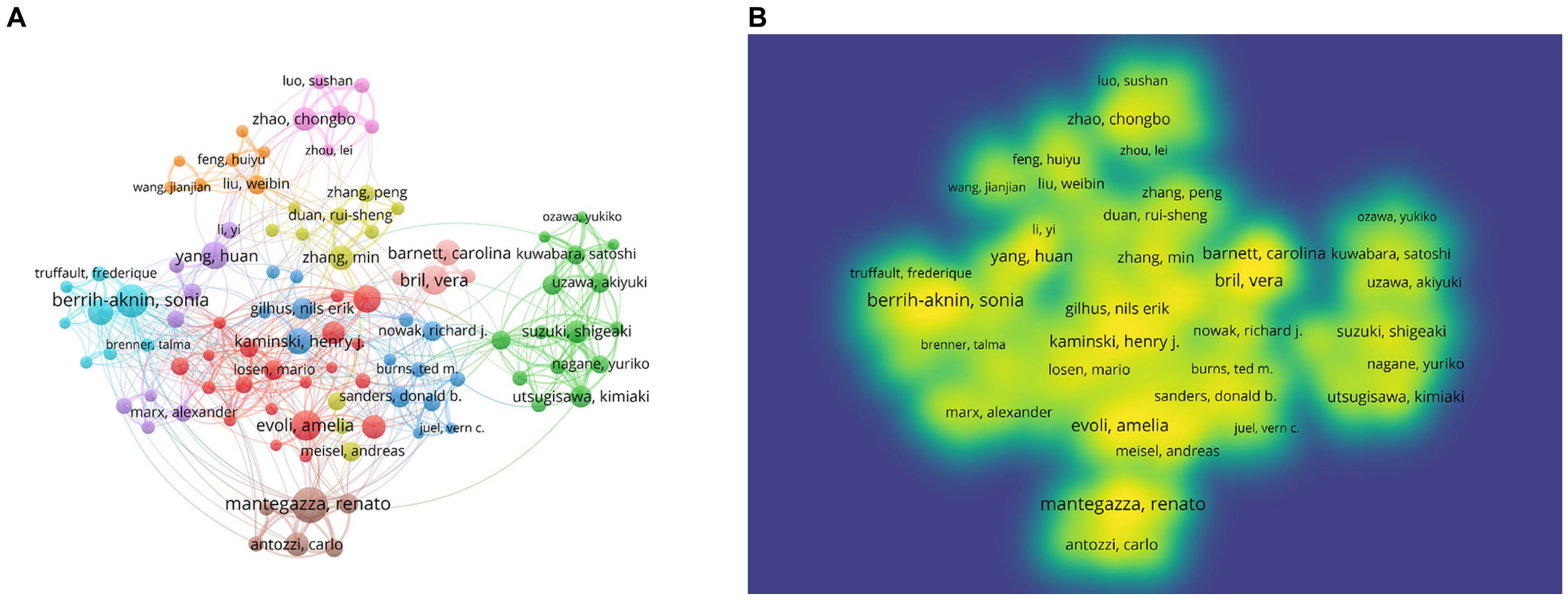
Network visualization of authors. **(A)** Visualization of authors divided into 10 clusters. **(B)** Density overlay visualization of the authors network.

One hundred five authors with at least 13 publications were included in the co-authorship analysis. The authors were divided into 10 clusters based on their co-authorship, and cooperation among authors was close, as shown in Figure 7A. Based on density visualization (Figure 7B), the central authors were Amelia Evoli, Renato Mantegazza, Vera Bril, Henry Kaminski, Sonia Berrih-Aknin, and Huan Yang. As shown in Table 4, the author with the highest NP was Renato Mantegazza (64), followed by Jan J. Verschuuren (63), and Amelia Evoli (61). Renato Mantegazza is an Italian researcher who has studied a wide range of myopathies and participated in a randomized clinical trial of efgartigimod for the treatment of generalized MG (8). Notably, the author with the highest NC was Amelia Evoli (4,216), followed by Jan J. Verschuuren (3,925) and Angela Vincent (3,865). Amelia Evoli is also from Italy, and by assessing cognitive dysfunction in muscle-specific tyrosine kinase antibody seropositive (MuSK+) passive transfer MG mice, she suggested that recognition memory in the perirhinal cortex of MuSK+ patients with MG could be affected (9). Her team also focuses on immunotherapy in MG (10) and detection methodologies of antibodies in MG (11).

**Table 4:** The top 10 most productive authors.

Rank	Author	Country	Np	Nc	H-index	Average citations
1	Mantegazza, Renato	Italy	64	2,628	28	41.06
2	Verschuuren, Jan J.	Netherlands	63	3,925	27	62.30
3	Evoli, Amelia	Italy	61	4,216	28	69.11
4	Vincent, Angela	England	59	3,865	33	65.51
5	Berrih-aknin, Sonia	France	56	3,044	32	54.36
6	Bril, Vera	Canada	53	1,671	20	31.53
7	Kaminski, Henry J.	the United States	48	2,232	21	46.50
8	Punga, Anna Rostedt	Sweden	41	772	18	18.83
9	Barnett, Carolina	Canada	41	737	16	17.98
10	Utsugisawa, K.	Japan	40	1,438	19	35.95
11	Antozzi, Carlo	Italy	40	1,628	22	40.70
12	Le Panse, Rozen	France	40	1,536	24	38.40

### 3.6 Co-citation analysis

**Figure 8:**
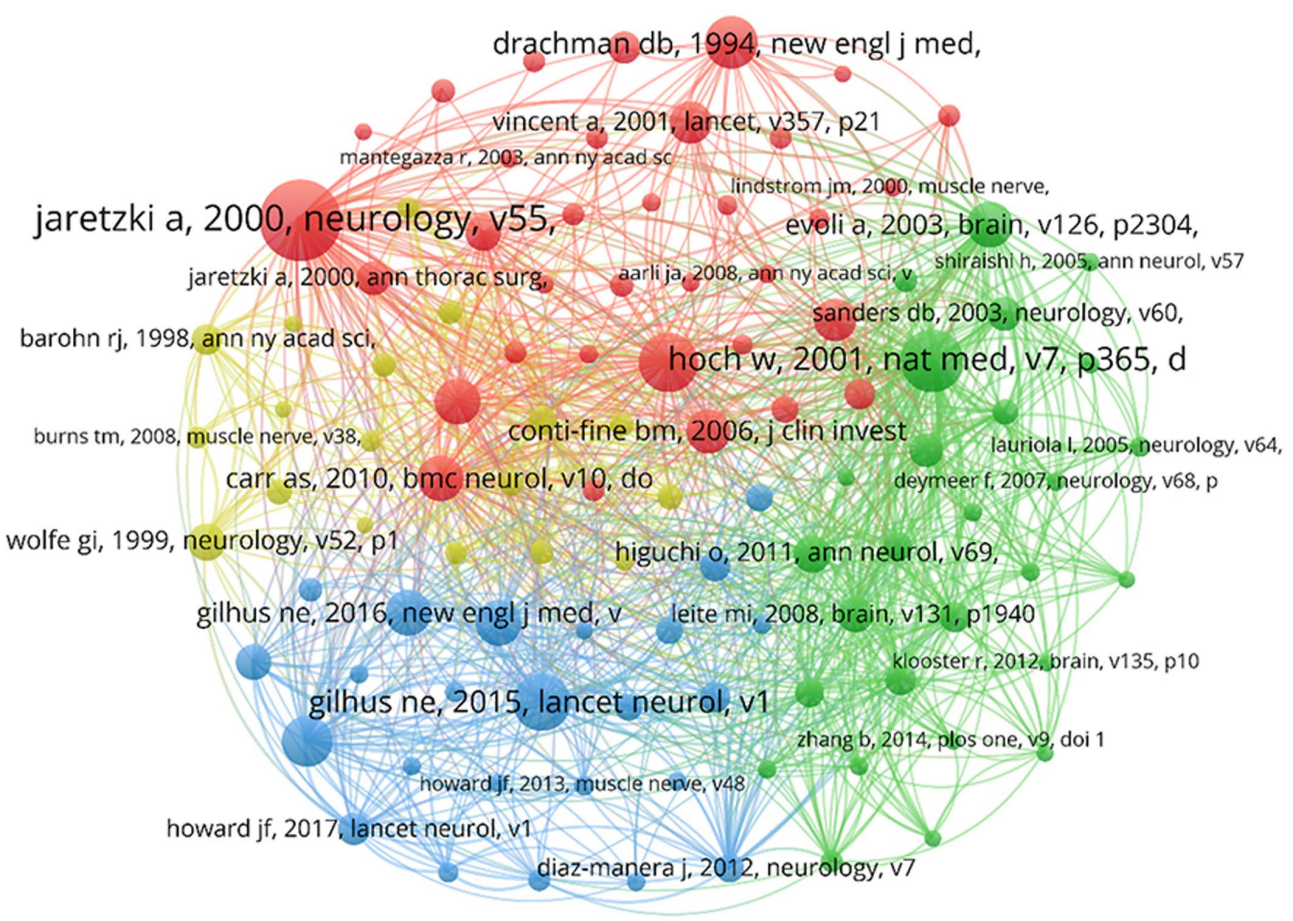
Network visualization of co-cited references.

The co-cited references were analyzed in VOSviewer, with a threshold of at least 65 co-citation counts, incorporating 103 publications (Figure 8). The NC serves as the most objective and direct measure of a publication’s significance (12). In the figure, the highly cited literature is divided into four clusters corresponding to four colors: red, yellow, blue, and green. The red cluster consists of 34 publications, primarily reviews, that laid the foundation for theory and were conducted before 2010. These contributions standardized the clinical treatment and scientific research of MG (13–15). The green category consists of 27 publications focused on the pathogenic autoimmune antibodies in patients with MG and the immunopathogenesis of MG, providing ideas for therapeutic drug development (16–18). The blue category consists of 24 publications focused on introducing clinical treatment protocols for MG and standardizing management of patients with MG (19–21). The yellow category consists of 18 publications focused on assessing the conditions of patients with MG, such as the design and application of scales, and establishing the clinical assessment and diagnostic criteria of MG (22–24).

### 3.7 Analysis of keywords

**Figure 9:**
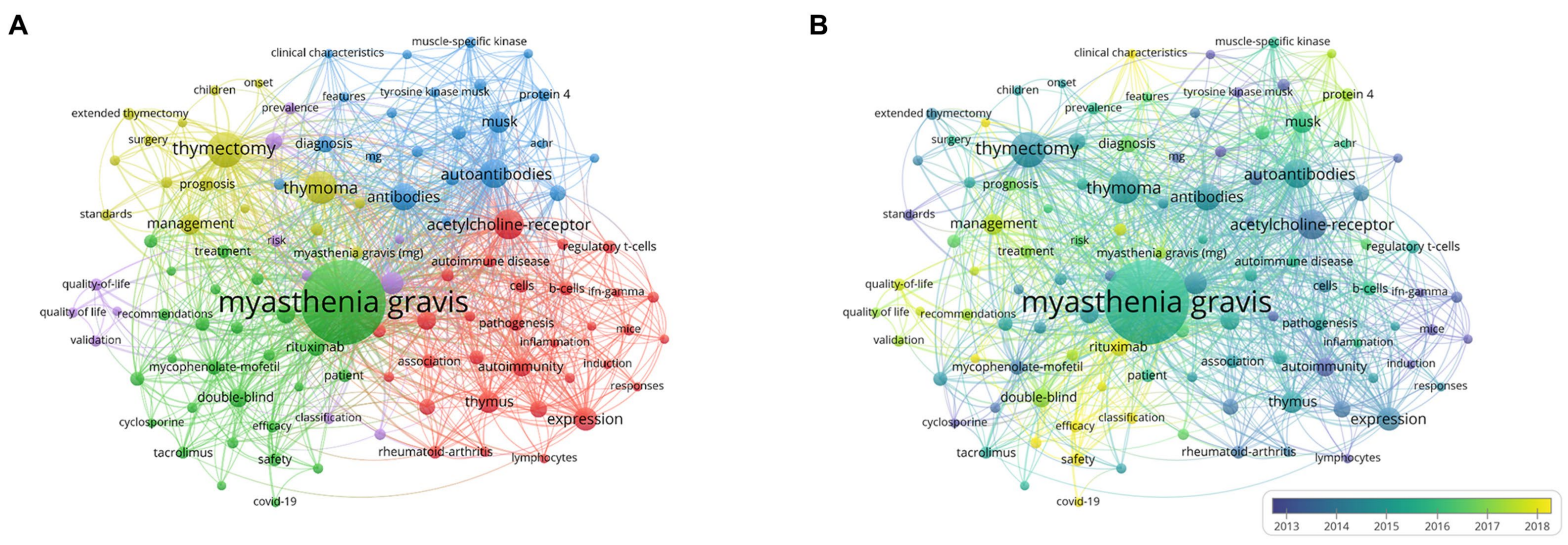
Network visualization of co-occurrence keywords. **(A)** Visualization of keywords divided into five clusters. **(B)** Time overlay visualization of the keywords network.

The co-occurrence analysis of keywords within the publications was performed using VOSviewer. A minimum occurrence of 40 was set, resulting in 123 keywords for visualization and analysis out of a total of 7,857 keywords (Figure 9A). Cluster analysis of keywords can identify popular research topics that can guide future directions. In the figure, larger circles represent a higher number of keyword occurrences. The lines connecting the circles indicate the frequency of co-occurrence, with different cluster colors signifying distinct research directions. The yellow cluster mainly focuses on thymectomy, featuring keywords such as “thymoma,” “thymectomy,” and “surgery.” The red cluster is associated with the pathogenesis of MG, including keywords such as “acetylcholine receptor,” “b-cells,” and “thymus.” The blue cluster, with keywords such as “autoantibodies,” “musk,” and “protein 4,” explores autoantibodies in patients with MG, revealing the relationship between autoantibodies and MG diagnosis. The green cluster is dominated by therapeutic aspects of MG, featuring keywords such as “rituximab,” “patient,” and “efficacy.” The purple cluster, with keywords such as “prevalence,” “epidemiology,” and “classification,” reflects its theme as epidemiology in MG. Representative terms in MG research, such as “myasthenia gravis,” “thymectomy,” “autoantibodies,” “acetylcholine-receptor,” and “disease,” constitute larger circles in each cluster. Figure 9B depicts the temporal characteristics of keyword co-occurrence from blue to yellow, representing the chronology from 2003 to 2022, with “rituximab,” “safety,” “classification,” “COVID-19,” and “clinical characteristics” as recent research hotspots.

**Figure 10:**
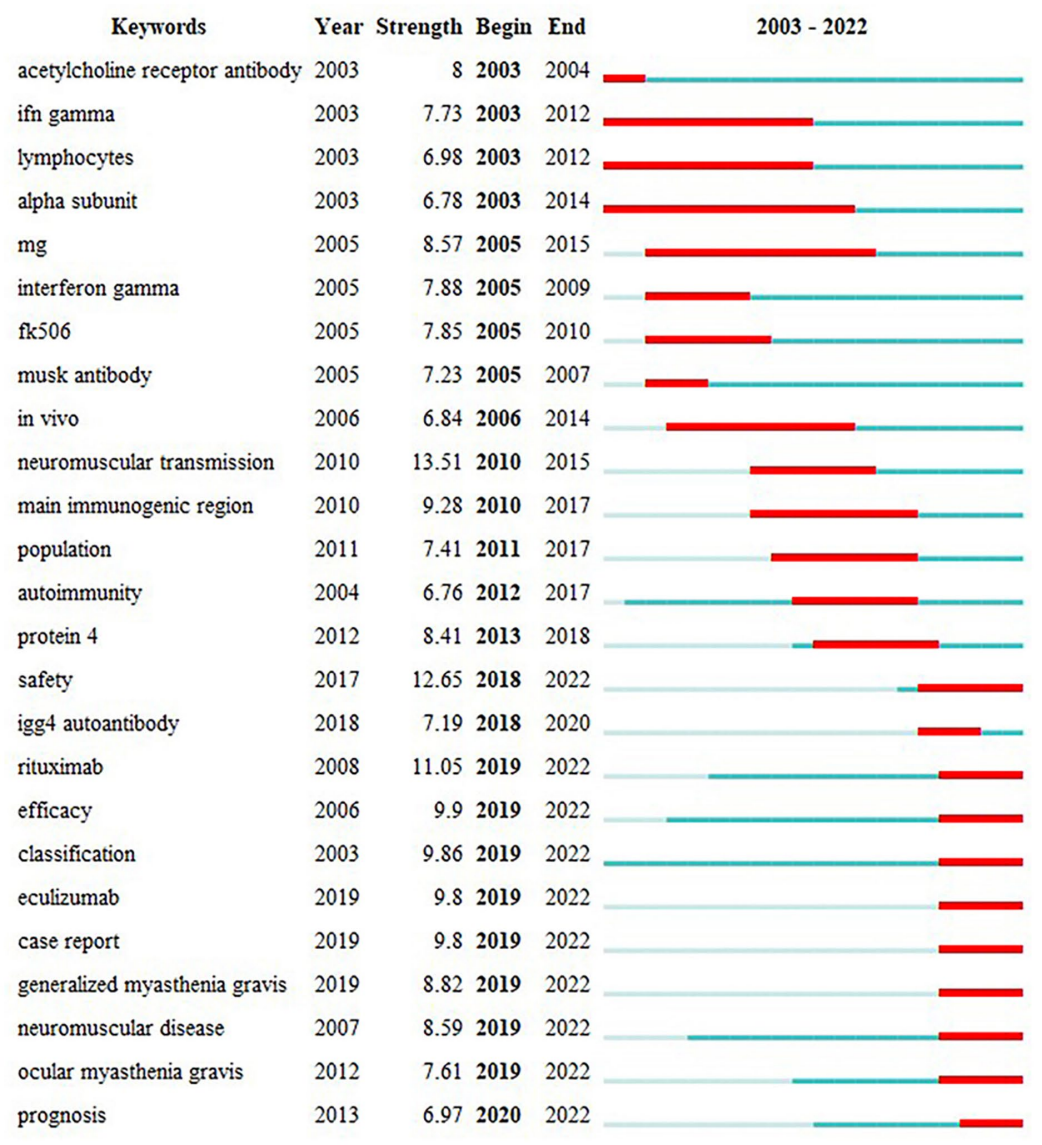
The top 25 keywords with the strongest citation bursts.

The keyword “outbreak analysis” highlights a sudden growth in keyword citations during a specific period, indicating research hotspots. Figure 10 presents the top 25 keywords with the strongest citation analysis. The red line indicates the time of the outbreak, whereas the blue line indicates the period. Keywords that burst earlier included “acetylcholine receptor antibody,” “IFN gamma,” “lymphocytes,” “alpha subunit,” and “neuromuscular transmission.” Keyword bursts in 2010–2015 that remained in the outbreak period were “safety,” “rituximab,” “efficacy,” “classification,” “eculizumab,” “case report,” “generalized myasthenia gravis,” “neuromuscular disease,” and “prognosis.” The shift in the burst keywords of MG reflects changing research interests in recent years, with “neuromuscular transmission” being the keyword with the highest strength (13.51), followed by “safety” (12.65) and “rituximab” (11.05). These represent current research hotspots.

### 3.8 Citation burst analysis

**Figure 11:**
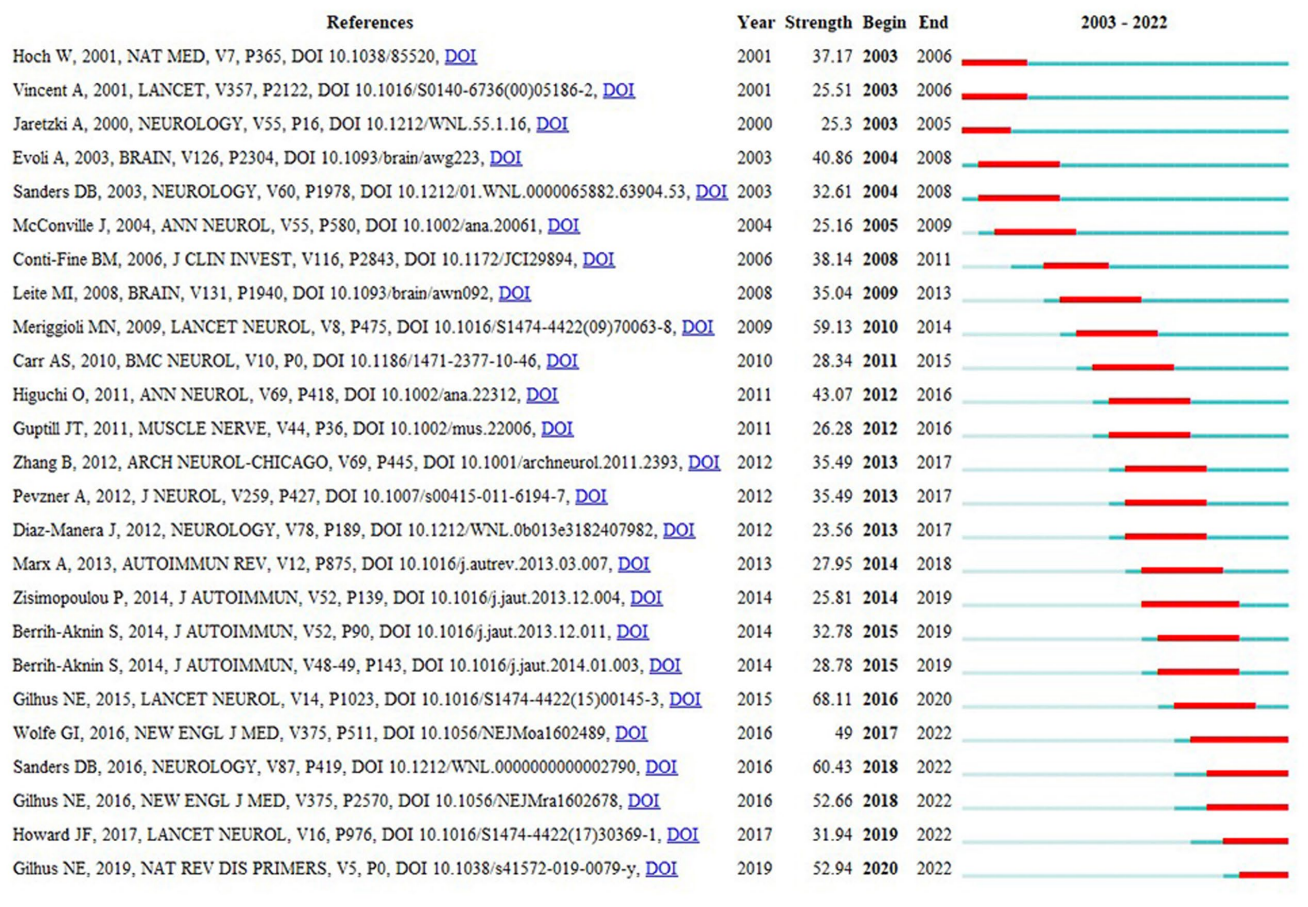
The top 25 references with the strongest citation bursts.

Figure 11 shows the citation burst analysis of the top 25 cited papers using CiteSpace. This analysis aimed to identify heightened interest during specific periods within the research area, accomplished by examining the temporal characteristics of the cited articles. Over the past two decades, the earliest articles contributing to the burst period were written by W. Hoch, A. Vincent, and A. Jaretzki. Hoch et al. explored the role of MuSK antibodies in MG pathogenesis (16), whereas Vincent et al. provided an overview of the diagnosis and treatment of MG (25). Jaretzki et al. suggested the standardization of clinical trials for MG (13). Publications during the burst phase included articles by G.I. Wolfe, D.B. Sanders, J.F. Howard, and N.E. Gilhus. Sanders et al. made significant contributions to the management of patients with MG (21). Gilhus and colleagues conducted an updated systematic review of MG (26, 27). Additionally, both G.I. Wolfe (28) and J.F. Howard (29) conducted randomized controlled clinical trials that provided substantial evidence supporting thymectomy and eculizumab as effective interventions for MG, respectively.

## 4 Discussion

In this study, we used bibliometrics to visualize publication trends in MG research from 2003 to 2022. We analyzed 3,610 eligible publications using VOSviewer and CiteSpace, exploring patterns in citations, contributing countries and regions, institutions, authors, journals, keywords, and co-citations. Specific bibliometric techniques, including burst hotspot, cluster, and keyword analyses, were conducted to determine the research status and future directions of MG research.

### 4.1 Knowledge base

The analysis of the change in annual NP indicated an overall upward trend. The analysis of the top 10 countries in terms of NP revealed that the USA had the highest NP and H-index, which indicates a higher quantity and quality of research in this field. The annual NP in China increased rapidly and exceeded that of the USA in 2014, indicating China’s rapid development in MG research. However, China’s lower citation ranking suggests a need for improved research quality and global impact. Intercountry cooperation is crucial, as seen in the visualization, with most countries collaborating closely. Countries at the periphery face challenges such as geographic distance, and they must strengthen cooperation with other countries, enhance research capacity, and delve into key scientific issues in the field.

Identifying institutions with a solid research base influences the selection of long-term work and cooperation among researchers. In a visual analysis of publishers and institutional collaborations, Oxford University and Duke University stand out, and research institutions must keep pace with these leaders to enhance capacity and explore deeper research. The time overlay analysis revealed that the University of Toronto, Capital Medical University, and Shandong University are up-and-coming institutions in MG research. Hence, it is prudent for government sectors to consider increasing the financial support for these institutions.

According to the visualization of co-authorship, cooperation among authors was close, enhancing research quality. Moreover, authors who play a crucial part in cooperations may have higher quality of research. The core authors of this cooperation were Amelia Evoli, Renato Mantegazza, Vera Bril, Henry Kaminski, Sonia Berrih-Aknin, and Huan Yang. Renato Mantegazza and Amelia Evoli are the most productive and most cited authors, respectively, showing significant influence of their study and outstanding contribution to this field of research. Researchers should read and refer to the publications of these scholars to identify the pivotal and renewed points of MG.

*Muscle & Nerve*, the *Journal of Neuroimmunology*, and *Neuromuscular Disorders* published the most articles, making them suitable outlets for the publication and dissemination of research in this field. Researchers could consider submitting their articles to these journals, and scholars can consult publications from these journals to obtain the latest information on MG.

In the co-citation science map, larger circles indicate articles with more citations, pointing to greater influence. The largest circle features Jaretzki et al.’s article, titled “Myasthenia Gravis: Recommendations for Clinical Research Standards” (13), published in *Neurology* and cited 918 times. This article addressed challenges in MG clinical trials, including refining the Myasthenia Gravis Foundation of America (MGFA) clinical classification and establishing the Quantitative Myasthenia Gravis (QMG) score, which remains a global scientific standard. Their efforts systematically organized critical details, facilitating uniformity in MG’s clinical research and management processes, explaining the enduring recognition and referencing of this article. The understanding of the MG knowledge base unfolds through the exploration of relevant research domains visualized in the science map generated by the VOSviewer. The four clusters in Figure 7 include topics ranging from the underlying pathogenesis to intricate clinical management and from foundational theoretical aspects to their practical applications. This comprehensive development is reflective of the extensive research conducted on MG from 2003 to 2022.

### 4.2 Research hotspot

Upon reviewing citations and keywords, the continuous improvement of precise therapeutic approaches for diverse patient profiles emerged as the hotspot of MG research. This can be categorized into three aspects: MG antibody-related subtype therapy, novel immunotherapeutic approaches, and thymectomy.

#### 4.2.1 Treatment strategies for different MG subtypes

The treatment options for MG include cholinesterase inhibitors, thymectomy, immunosuppressive or immunomodulatory medications, and plasma exchange. However, the increasing prevalence of MG demands more effective therapeutic approaches. The keyword “classification” stands out in recent research, especially during the burst period. Notably, an article published in 2015 in *Lancet Neurology* (19) covering autoantibodies, epidemiology, clinical presentation, and comorbidities holds the highest burst strength (68.11) in reference citation burst detection. It summarizes potential treatments for MG and suggests a future research direction of exploring new immunosuppressive drugs and drug combinations tailored to MG subgroups. This article plays a crucial role in subgroup classification and provides valuable insights for future research in MG. The emphasis on burst keywords and references underscores researchers’ keen interest in MG with different antibodies, emphasizing the crucial role of autoantibodies in MG diagnosis, understanding patient features, and personalizing treatment approaches. Most patients with MG have antibodies targeting AChRs, with fewer having antibodies against MuSK or LRP4 (30). A multicenter study showed that patients with antibodies against LRP4 and/or agrin exhibit more generalized symptoms (69%) than antibody-negative patients, but most of them responded favorably to standard MG therapy (31). This showcases how studying the clinical characteristics of MG with specific antibodies can significantly improve diagnosis and management. MuSK, as an antibody, differs from classic AChR antibodies, impacting clinical manifestations and treatment responses, thus posing challenges to accurate diagnosis and management. MuSK antibody-positive MG is prevalent among females (32), affecting muscles not typically weakened in non-MuSK antibody-positive MG, with increased respiratory weakness in this subgroup (33). Cholinesterase inhibitors often yield unsatisfactory results; therefore, early rituximab administration is recommended (34, 35). Future research will likely focus on treatments for patients with MG who have other specific autoimmune antibodies.

#### 4.2.2 Novel immunotherapeutic approaches

In 1996, a soluble recombinant form of human complement receptor 1 was demonstrated as an additional therapeutic approach for MG (36), suggesting that complement inhibition may be a potential therapeutic approach for MG. A phase 3, randomized, double-blind, placebo-controlled, multicenter study evaluating the efficacy and safety of eculizumab in AChR-positive refractory generalized MG was conducted during the burst period (29). Eculizumab, the first complement-specific drug clinically used for the treatment of paroxysmal nocturnal orphan disease and hemoglobinuria, has bolstered confidence in therapeutic complement inhibition (37) and has gained market approval in countries such as the USA, Japan, and China. As reported in a retrospective study, patients treated with eculizumab were more likely to achieve minimal manifestations than those treated with rituximab, but the risk of crisis was not reduced (38). Eculizumab has also been confirmed to benefit refractory AChR-MG in a real-world experience (39), but one patient reported acute worsening after discontinuation of eculizumab (40). Moreover, the optimal duration of treatment remains unclear. Therefore, further studies are needed on safety and treatment duration. Research to identify biomarkers predicting the response to eculizumab is also necessary. Eculizumab was also a keyword in the burst period, indicating attention to selective immunosuppressants in MG treatment. Ravulizumab is a monoclonal antibody complement inhibitor approved by the Food and Drug Administration (FDA) in April 2022 for AChR-Ab-generalized MG. A phase 3, randomized, double-blind, placebo-controlled, multicenter trial in patients with AChR-Ab-generalized MG indicated improved clinical outcomes for patients treated with ravulizumab, and the drug was well tolerated (41). The latest pharmacokinetics and pharmacodynamics research based on the data from this phase 3 study supports dosing every 8 weeks for immediate, complete, and sustained inhibition of terminal complement C5, and it could reduce the burden on patients (42).

Targeting the neonatal fragment crystallizable receptor (FcRn) is another novel therapeutic approach considered for generalized MG that has failed standard treatment. Meanwhile, efgartigimod, a human IgG1 antibody that reduces IgG recycling and increases IgG degradation by outcompeting endogenous IgG binding (43), was approved by the FDA in July 2022 for the treatment of AChR-Ab-generalized MG in adult patients. A phase 3, randomized, double-blind, placebo-controlled trial in generalized MG revealed better clinical improvement in the efgartigimod arm than in the placebo arm, whereas most patients had AChR-MG (44). A meta-analysis found that anti-FcRn has an advantage in improving the QMG score than complement treatments in patients with generalized MG, and they also proposed the high probability of efgartigimod and rozanolixizumab being the most effective treatment in generalized MG (45).

Rituximab, a keyword with high citation strength and more recent citations, is a monoclonal antibody directed against CD20 antigen on B cells that has been used for years. The clear benefit of refractory MuSK antibody-positive MG has been demonstrated; however, its efficacy in AChR-MG remains controversial (46). A systematic review of 13 studies proposed that the small number of patients with AChR-MG in previous studies may have caused bias in efficacy evaluation and suggested that dosages for different subtypes of MG should be considered in future studies (47). In addition, due to the off-label use of rituximab and the inherent risk of infection associated with continuous B-cell depletion, safety is a crucial factor and should be the focus. For example, infection is the most common side effect of this treatment. A retrospective study of adverse events in treatment of refractory MG with rituximab found that infection was associated with hypogammaglobulinemia and proposed that a standardized monitoring scheme of IgG is necessary (48). To ensure the safety of rituximab in patients with MG and enhance risk control, high-quality randomized controlled trials with large samples should be conducted.

#### 4.2.3 Thymectomy

Thymectomy is inevitable in thymomatous MG (49). Moreover, a multicenter, randomized, single-blind trial comparing thymectomy combined with prednisone to prednisone alone, published in the *New England Journal of Medicine* in 2016 (28), provided a conclusive outcome that thymectomy benefits patients with MGFA clinical class II to IV disease with non-thymomatous AChR-MG. These authors extended the study up to 2 years to investigate the durability of the treatment associated with thymectomy, published in 2019, further affirming the advantages of thymectomy and reversing the treatment decision trends for this procedure (50). Both aforementioned studies were still in the burst period, indicating sustained interest in thymectomy studies. However, the prognosis of thymectomy in certain outcomes can be observed in the worsening or relapse of MG, even after thymectomy. This may be associated with patient heterogeneity, autoantibody profiles, thymic pathology, operation-associated factors, perioperative care, and disease conditions, among other factors (51). To better guide the decisions between conservative treatments or surgery, the pivotal mechanisms of clinical thymectomy in MG need to be elucidated, which may be a promising topic for future research.

### 4.3 Advantages and limitations

The present research has some novel contributions to this field. First, this is the first bibliometric study that not only systematically analyzed research of MG but also paid special attention to research hotpots, especially MG treatment. Therefore, it will be beneficial to scholars who are interested in this subject as well as neurologists who want to catch up with recent advances in a visual manner. Second, based on our study, we proposed some rational recommendations for potential project sponsor and related government sector. Resource integration could be further enhanced, which may stimulate the development of MG research.

This bibliometric analysis has several limitations. First, the NC of recently published articles may not fully reflect their quality, potentially introducing bias into our qualitative assertions (52). Second, as this study exclusively included publications in English, articles in other languages were omitted, potentially introducing bias.

### 4.4 Conclusion

Using VOSviewer and CiteSpace, this study has dynamically clarified the trajectory of MG research, revealing its developmental nuances, hotspots, and future trends. Notably, the USA had the highest NP and NC. Nevertheless, there is potential for more collaboration among countries and institutions. Current MG research shows enthusiasm, especially for individualized treatments based on subtypes, novel immunotherapeutic approaches, and thymectomy. These facets collectively shape ongoing and future research into the intricacies of MG.

## Data availability statement

The original contributions presented in the study are included in the article/supplementary material, further inquiries can be directed to the corresponding author.

## Author contributions

YT: Data curation, Writing – original draft, Writing – review & editing, Formal Analysis, Investigation, Visualization. QS: Data curation, Writing – original draft. SP: Data curation, Writing – review & editing. LM: Writing – review & editing. RF: Data curation, Writing – review & editing. AX: Investigation, Writing – review & editing. SL: Writing – review & editing. YY: Writing – review & editing. WC: Writing – review & editing. JN: Conceptualization, Writing – review & editing. WZ: Conceptualization, Writing – review & editing.
